# Diet and the development of the human intestinal microbiome

**DOI:** 10.3389/fmicb.2014.00494

**Published:** 2014-09-22

**Authors:** Noah Voreades, Anne Kozil, Tiffany L. Weir

**Affiliations:** Department of Food Science and Human Nutrition, Colorado State UniversityFort Collins, CO, USA

**Keywords:** enterotype, gut microbiome, aging, dietary patterns, colonization

## Abstract

The important role of the gut microbiome in maintaining human health has necessitated a better understanding of the temporal dynamics of intestinal microbial communities as well as the host and environmental factors driving these dynamics. Genetics, mode of birth, infant feeding patterns, antibiotic usage, sanitary living conditions and long term dietary habits contribute to shaping the composition of the gut microbiome. This review focuses primarily on diet, as it is one of the most pivotal factors in the development of the human gut microbiome from infancy to the elderly. The infant gut microbiota is characterized by a high degree of instability, only reaching a state similar to that of adults by 2–3 years of age; consistent with the establishment of a varied solid food diet. The diet-related factors influencing the development of the infant gut microbiome include whether the child is breast or formula-fed as well as how and when solid foods are introduced. In contrast to the infant gut, the adult gut microbiome is resilient to large shifts in community structure. Several studies have shown that dietary changes induce transient fluctuations in the adult microbiome, sometimes in as little as 24 h; however, the microbial community rapidly returns to its stable state. Current knowledge of how long-term dietary habits shape the gut microbiome is limited by the lack of long-term feeding studies coupled with temporal gut microbiota characterization. However, long-term weight loss studies have been shown to alter the ratio of the Bacteroidetes and Firmicutes, the two major bacterial phyla residing in the human gastrointestinal tract. With aging, diet-related factors such as malnutrition are associated with microbiome shifts, although the cause and effect relationship between these factors has not been established. Increased pharmaceutical usage is also more prevalent in the elderly and can contribute to reduced gut microbiota stability and diversity. Foods containing prebiotic oligosaccharide components that nurture beneficial commensals in the gut community and probiotic supplements are being explored as interventions to manipulate the gut microbiome, potentially improving health status.

## IMPORTANCE OF THE GUT MICROBIOME

The consortium of single-celled organisms residing in our intestines, the gut microbiome, is rapidly emerging as an important determinant of health. Deterrents to proper bacterial colonization in early life are hypothesized to contribute to food sensitivities, allergic reactions, Type I diabetes, and other autoimmune disorders (Kelly et al., 2007). Association of the microbiome to autoimmune diseases has been explained by the “hygiene hypothesis,” which suggests that the absence of a robust microbiome results in defects in development and regulation of the immune system, resulting in a lack of immune tolerance (Okada et al., 2010; Rook, 2012). Later in life, strong evidence supports an important role for intestinal microbiota in weight regulation via contributions to dietary energy harvest and appetite control (Tilg and Kaser, 2011). The gut microbiome has also been implicated in the pathology of several intestinal inflammatory diseases as well as in the development of colorectal, gastric, and prostate cancers and cardiometabolic disorders (Sekirov et al., 2010). Mechanisms giving rise to these conditions include the production of genotoxins by bacterial pathogens, microbial metabolism of dietary components to produce carcinogenic compounds, and inciting local and systemic inflammatory cascades that result in chronic low grade inflammation and damage to affected tissues and organs.

While a dysbiotic microbiota can cause disease, a healthy microbial community is vital to assist the host in maintaining optimal wellness. Thus, there is a need to understand the factors that shape and alter the microbiome throughout the lifespan of an individual. Numerous elements, encompassing environmental exposures, genetics, and other inherent host factors, contribute to the initial colonization of the microbiome in infants and to the subtle shifts that occur in adults, occasionally culminating in microbial decline as observed in frail and unhealthy elderly individuals (Koenig et al., 2011). However, none of these factors may be as important in the development of the microbiome as diet. In this review we will present evidence for the importance of diet in initial colonization events and in determining the composition of a stable adult microbiome. Factors such as malnutrition and pharmaceutical interventions on the aging gut will also be reviewed. Finally, we will discuss potential interventions, including dietary changes that can be used to alter the intestinal microbial community.

## EARLY MICROBIAL COLONIZATION AND ESTABLISHMENT

**FIGURE 1:**
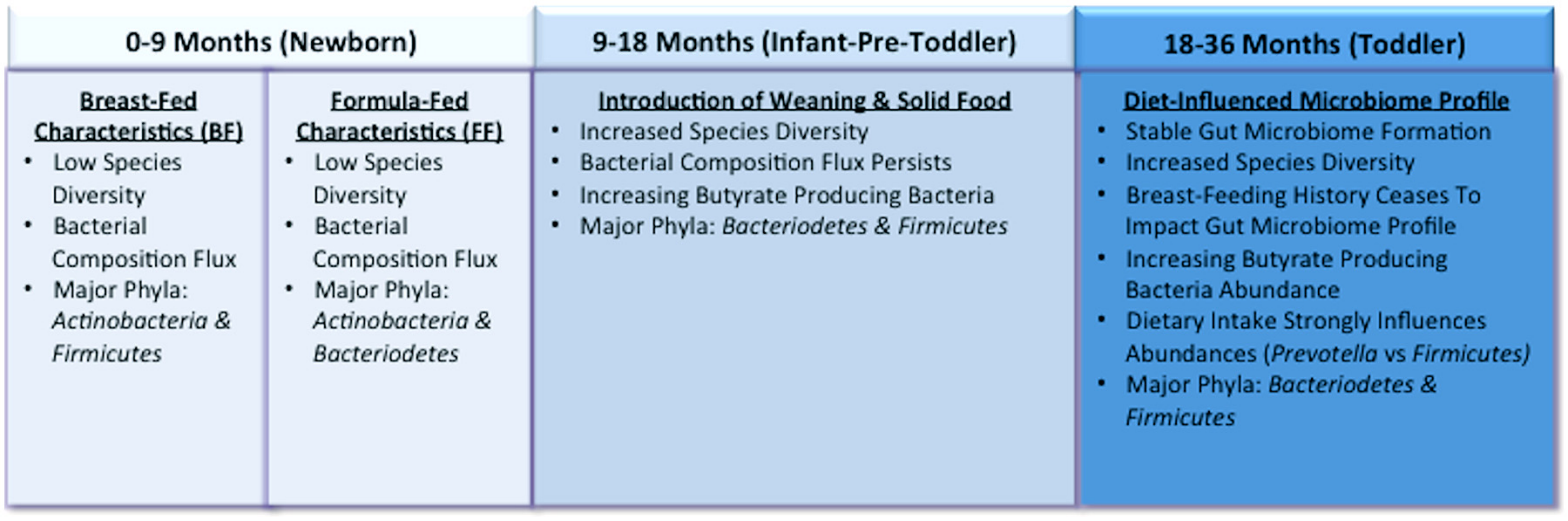
**Representation of the infant gut microbiome development from birth to 3 years of age.** By 3 years old, toddler’s microbiomes are similar to that in adults and long-term dietary patterns are beginning to establish.

The infant gut is thought to be sterile at birth, although some new research characterizing the placental microbiome challenges that assumption (Aagaard et al., 2014). After birth initial colonization and early establishment of the infant gut is influenced by whether delivery was vaginal or caesarean, feeding patterns, sanitary conditions, and antibiotic administration (Marques et al., 2010). The relative importance of these factors on the long-term structure of the intestinal microbial community and associated health outcomes is still debated. It stands to reason that with constant exposure between the microbiome and food components that diet is one of the primary drivers shaping the changes that occur during infancy and the structure of the adult microbiome that eventually establishes. This section will focus on the diet’s role in shaping the infant gut microbiome from birth to ∼3 years of age. Specifically, the following topics will be explored in detail: (1) the influence of breast vs. formula-feeding in initial colonization, (2) changes related to beginning of weaning and introduction of solid foods, and (3) factors contributing to a stable gut microbiome profile (**Figure 1**).

### BREAST vs. FORMULA FEEDING

Following birth, the infant gut microbiome is characterized by low-species diversity and high rates of bacterial flux until ∼2 or 3 years old (Bergström et al., 2014). Facultative anaerobic bacteria including *Staphylococcus, Streptococcus, Escherichia coli* and *Enterobacteria* are thought to be the first colonizers of the gut. Their purpose is to consume oxygen and create an environment for obligate anaerobes to thrive (Palmer et al., 2007; Jost et al., 2012). These are later replaced by facultative anaerobes that dominate the gastrointestinal tract, primarily Actinobacteria and Firmicutes (Turroni et al., 2012). This change in dominant taxa representation can be attributed to the introduction of breast or formula-feeding, signifying the first diet-related colonization event in the infant gut microbiome (Harmsen et al., 2000; Jost et al., 2012). In breast-fed infants, the dominant Actinobacteria are represented by *Bifidobacterium* species, specifically, *B. breve*, *B. longum*, *B. dentium*, *B. infantis*, and *B. pseudocatenulatum* (Harmsen et al., 2000; Jost et al., 2012). The Firmicutes phylum is represented principally by lactic acid bacteria such as *Lactobacillus* and *Enterococcus* as well as *Clostridium* species (Turroni et al., 2012; Bergström et al., 2014). More than 700 species of bacteria have now been identified in human colostrum and breast milk, including multiple species of lactic acid bacteria as well as species typically colonizing the oral cavity of infants (Cabrera-Rubio et al., 2012). While this may contribute to the intestinal community of breastfed infants, it is still unclear whether the composition of species in breast milk is driven by transfer from infant to mother. The chemical composition of breast milk does influence the gut microbiome through supplying unique oligosaccharides that are selectively utilized by *Bifidobacterium spp.* (Turroni et al., 2012).

There are conflicting reports regarding differences in the relative abundance of these bacteria between breast and formula fed infants. Many studies have reported that formula-fed infants display dominance of *Bifidobacterium spp.* similar to what has been observed in breastfed infants (Harmsen et al., 2000; Fallani et al., 2010, 2011). However, another study reported approximately double the count of *Bifidobacterium* in breast fed infants compared to those fed formula (Bezirtzoglou et al., 2011). Formula feeding was also associated with higher levels of *Atopobium* (Bezirtzoglou et al., 2011); which corroborated reports by Fallani et al. (2010), although they only noted *Atopobium* increases in formula fed infants delivered by Cesearean section or whose mother’s had been administered antibiotics. Higher numbers of *Bacteroides spp*. as well as members of the Enterobacteriaceae have also been reported in formula-fed infants (Harmsen et al., 2000; Fallani et al., 2010). Despite significant evidence that *Bifidobacterium* is an important early colonizer in neonates, Palmer et al. (2007) reported that *Bifidobacterium* was not present in significant amounts in the infant gut (Palmer et al., 2007). However, it is important to highlight that within their cohort, there was a mixture of breast and formula-feeding, antibiotics were provided to infants and a small subset required specialized hospitalization.

The variability reported with regard to *Bifidobacterium* abundance could be driven by differences in infant formula composition. Formulas supplemented with the prebiotics galacto-oligosaccharide (GOS) and fructo-oligosaccharide (FOS) may account for high levels of *Bifidobacterium* found in many formula-fed infants (Marques et al., 2010; Oozeer et al., 2013). A recent review discusses evidence supporting GOS and FOS supplementation effects on the gut (Oozeer et al., 2013). Infant gut microbial populations provided with either human breast milk or prebiotic supplemented infant formula had similar levels of *Bifidobacterium;* whereas gut microbial populations of infants given traditional formula was reported to have about 20% fewer *Bifidobacterium* (Knol et al., 2005). Additionally, the species composition of *Bifidobacterium* was similar between infants given human breast milk and those on prebiotic supplemented formula. However, traditional formula fed infants had markedly different gut microbial communities and even the specific *Bifidobacterium* species differed with higher relative abundances of *B. cantenulatum* and *B. adolescentis,* which are typically represented in adult populations. Another potential explanation for the variation in studies reporting bacterial abundances, particularly with regard to breast-feeding could be due to differences in the maternal-diet (Cabrera-Rubio et al., 2012). Characterization of the placental microbiome suggests that it is colonized by the mother’s oral microbiome (Aagaard et al., 2014). Another recent study showing that pre and post-natal maternal consumption of a high fat diet, independent of obesity in the mother, resulted in dysbiosis of the infant gut in a primate model (Ma et al., 2014). Together, these studies suggest that maternal diet may play a significant but previously unrecognized role in determining early colonization and establishment of the infant microbiome. Conduct of randomized trials in which the maternal diet is controlled or large-scale cross-sectional studies of pregnant mothers adhering to different diets (Western, vegetarian, gluten-free, etc) are necessary to further develop this hypothesis.

### WEANING AND THE SHIFT TOWARD AN ADULT MICROBIOME

Around the age of 1–2 years old, the infant gut microbiome undergoes its second shift and the stable adult microbiome begins to emerge, further supporting the significant role of the diet in influencing the microbial community (De Filippo et al., 2010; Bergström et al., 2014). One study reported that although there were differences in the microbiome pre- and post-weaning, the impacts of earlier colonization events (delivery mode, formula or breastfed, etc.) were still apparent (Fallani et al., 2011). Another study comparing Italian vs. African children’s gut microbiomes showed that after weaning and solid foods were introduced there was a significant diet-related shift in the gut microbiome profiles. Prior to the introduction of their respective Western or African diets, the children across both populations that were still breast-feeding clustered together and had similar *Bifidobacterium* species dominance. Only children who were already weaned reliably clustered together into distinct geographic groupings. This study reinforced two important points related to dietary drivers of the gut microbiome development in children. First, breast-feeding, regardless of duration supports a specific bacterial state that is unique and markedly different from that observed in individuals consuming solid foods. Second, once solid foods are introduced, its role in shaping long-term gut microbiome profiles is so strong that individual’s cluster based on diet type over other environmental and physiological factors (De Filippo et al., 2010).

A similarly significant shift was reported by Bergström et al. (2014) in a 3 year Danish study with a cohort of 330 infants. They reported that between 9 and 18 months, the infant gut bacterial abundances changed drastically with the introduction of solid foods. Specifically, *Bacteroidetes-*related species increased. Whereas *Bifidobacterium* and *Lactobacillus* species and Enterobacteriaceae declined, various species within Firmicutes phylum were also reported to increase. This bacterial taxa shift is logical given that breast and/or formula-feeding has ceased, depleting the primary fuel source for these bacteria. In addition, butyrate producing bacteria such as *Clostridium leptum* group, *E. halli*, and *Roseburia* species increased. Typically, butyrate producing bacteria are responsible for the breakdown of otherwise indigestible complex plant polysaccharides and resistant starches. Anecdotally, this study found that the longer infants were breast and/or formula-fed, the lower their levels of butyrate producing bacteria. Additionally, more and different species begin to appear with introduction of solid foods (Koenig et al., 2011; Bergström et al., 2014).

### EMERGENCE OF A STABLE GUT PROFILE

From 18 to 36 months, the infant gut microbiome undergoes its final significant shift to a more stable microbial profile composed primarily of the bacterial phyla Bacteriodetes and Firmicutes. This shift represents a temporal change that can be attributed to the continued influence of a varied solid food diet (De Filippo et al., 2010; Koenig et al., 2011; Bergström et al., 2014). The earlier that solid food is introduced into the diet, the more quickly the gut microbiome begins to resemble a stable adult-like microbiome (Bergström et al., 2014). The specific proportion of Firmicutes and Bacteroidetes is strongly influenced by diet. This was best demonstrated in the previously discussed work by De Filippo et al. (2010) where the distinct microbial signatures of the two groups of children were indicative of their respective dietary habits. The most compelling evidence for this was the dominance of *Prevotella*, capable of digesting complex plant polysaccharides, in African children and its absence in Italian children. Similar diet-driven influences were reported in a detailed temporal study of a single infant. This study demonstrated that introduction of peas, formula, and other solid foods led to an emerging co-dominance between Firmicutes and Bacteroidetes, with the increase in Bacteroidetes potentially resulting from requirements for the breakdown of newly introduced plant polysaccharides (Koenig et al., 2011). The previously mentioned emergence of a stable gut microbiome can be substantially derailed if the infant experiences either severe acute malnutrition or moderate acute malnutrition. Emerging research is demonstrating that either of these malnutrition states has the potential to significantly alter the development of a healthy gut microbiome profile, regardless of diet-based interventions (Subramanian et al., 2014). These recent findings not only support a link between diet and the development of a particular gut microbiota and microbiome, but illustrate that nutrient quantity can impact development too.

## THE ADULT MICROBIOME

The typical adult intestinal microbiome is primarily comprised of approximately six or seven different bacterial phyla, of which Bacteroidetes and Firmicutes dominate (Eckburg et al., 2005). Less abundant phyla can include Proteobacteria, Verrucomicrobiota, Actinobacteria, and *Euryarchaeota*. A recent study followed changes in the microbiome of 37 adults for up to 5 years and reported that ∼60–70% of the bacterial strains present remained unchanged over the course of the study and that the most stable members of the microbiome tended to be the most abundant (Faith et al., 2013). They also observed that at the phyla level, Bacteroidetes and Actinobacteria populations were less susceptible to perturbations whereas Firmicutes and Proteobacteria were significantly less stable. These results are fairly consistent with findings from an earlier study utilizing a microarray-based approach to determine molecular taxonomy and which followed a smaller cohort over a longer period of time (Rajilic-Stojanovic et al., 2013). Both studies reported that the taxa present in an individual remain fairly consistent over time, although the relative abundances of these taxa were subject to change. However, data from Rajilic-Stojanovic et al. (2013) suggests that larger fluctuations occur between samples taken at longer intervals while Faith et al. (2013) report the opposite trend, with larger fluctuations occurring in samples taken over shorter periods of time compared to those that are temporally farther apart. Despite this resilience, there is evidence that the diet shapes the relative abundance of dominant phyla and populations of specific bacterial groups are influenced by the composition of macronutrients consumed.

### DIET-DRIVEN ENTEROTYPES

There have been numerous attempts to identify a “core” microbiota, usually defined as bacterial taxa that are shared between 95% of individuals tested (Huse et al., 2012). Identification of a core microbiome is important for defining a “normal” healthy state from which major variations may indicate a dysbiotic system that can result from or contribute to disease development. One barrier to defining an intestinal core microbiome has been the vast degree of variation between individuals. The microbial communities identified in samples collected from an individual over time are more similar to each other than microbial communities between two individuals, although related persons share more bacterial strains than unrelated individuals (Palmer et al., 2007; Yatsunenko et al., 2012; Faith et al., 2013). Although a consensus for what constitutes a core gut microbiome has been elusive, one report suggested that an international cohort of 39 individuals could be assigned to one of three distinct clusters or “enterotypes” based on metagenomic sequences (Arumugam et al., 2011). They found that each cluster was dominated by a particular bacterial genus (*Bacteroides*, *Prevotella*, and *Ruminococcus*) with positive or negative associations with a number of other genera in the community. They also reported that each cluster was enriched for specific gene functions that reflected different microbial trophic chains. Two of the three original enterotypes, *Bacteroides*, and *Prevotella*, were later confirmed and long term dietary patterns were identified as the primary predictor of an individual’s enterotype (Wu et al., 2011). The *Bacteroides* enterotype was associated with a Western-type diet high in proteins and fat, while the *Prevotella* enterotype was associated with plant fiber consumption. These enterotypes appear to be extremely stable, and several studies utilizing short-term interventions failed to result in a change in the assigned enterotype of participants (David et al., 2014; Roager et al., 2014).

The existence of enterotypes provided a convenient way of classifying individuals based on their fecal microbiota (although some argue a more appropriate term would be “faecotype”) and speculation has begun as to whether enterotypes can be used as a predictor of long term health risks. However, a microbial survey of several body sites, including stool, conducted with more than 200 individuals showed only minimal segregation into the *Bacteroides* and *Prevotella* enterotypes rather than the distinct and well separated clusters previously reported (Huse et al., 2012). These discrepancies could be due to the fact that the method for assigning enterotypes is not consistent across studies. An analysis of archived 16S sequences also showed that enterotype determination is sensitive to clustering methods and distance metrics used and that there is a continuum of *Bacteroides* abundances across samples rather than a bimodal distribution (Koren et al., 2013). These studies suggest that the enterotype concept is not be as clear cut as previously believed, and that standard methods for defining enterotypes should be developed and employed before they can be meaningfully tied with clinical outcomes.

### LONG TERM DIETARY PATTERNS AND THE MICROBIOME

**FIGURE 2:**
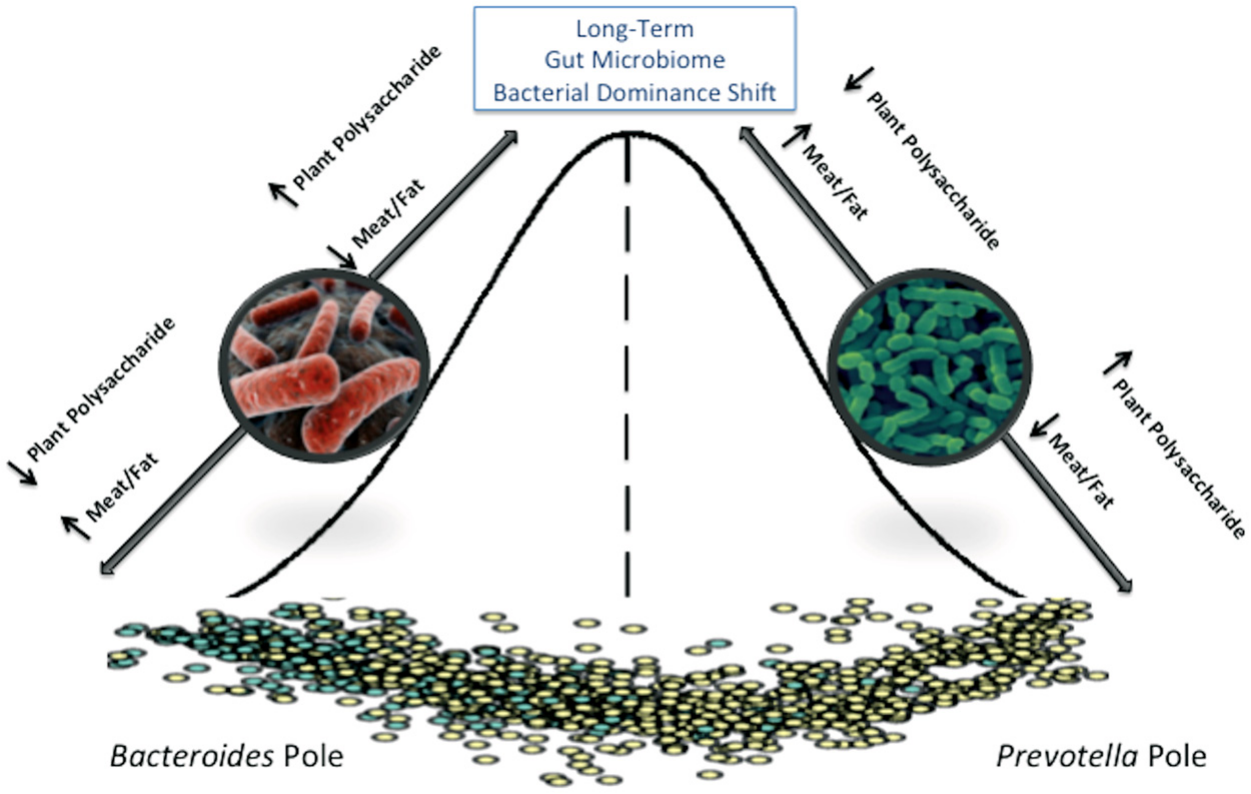
**The adult gut microbiome is characterized as existing in a steady state that requires a major disturbance to permanently alter that state.** Short-term diet interventions may transiently alter the gut microbiome community structure, but long-term diet changes are required to shift to a new steady-state.

Whether enterotypes truly exist or not, it is clear that diet is an important factor in shaping the microbiome (**Figure 2**). In addition to the divergence in microbial composition of Italian children and those from Burkina Faso shortly after weaning (De Filippo et al., 2010); other studies have shown microbiota segregation of individuals from Malawi, Venezuela, and the United States (Yatsunenko et al., 2012); children from Bangladesh and the United States (Lin et al., 2013), and between rural Africans and African Americans (Ou et al., 2013) that are at least partially diet-driven. In the Yatsunenko et al. (2012) study, metagenomic sequences revealed that enzyme classifications associated with protein degradation and bile salt metabolism were enriched in samples from the U.S. population where protein and fat consumption is high. Conversely, glutamate synthase and starch degrading enzymes were more abundant in the Amerindian and Malawian samples; consistent with protein poor diets of corn and cassava. This has been further demonstrated in a recent study of the diversity and metabolism of the microbiome of a Tanzanian hunter gatherer tribe, the Hadza. This study identified differences in the microbiome between the sexes which were consistent with their division of labor with regard to foraging (Schnorr et al., 2014). They also have many bacterial species associated with fermentation of plant-based fibers and are completely deficient in *Bifidobacterium*, which was hypothesized to result from the lack of meat and dairy in the diet; substrates that allow these bacteria to continue to colonize Westerners into adulthood. Although comparative studies between populations with different diets has been useful in identifying how dietary patterns shape the microbiome, these studies have utilized international cohorts that introduce confounding factors such as extreme differences in culture and environment. Relatively few studies have been conducted that examine the effects of diet on homogenous populations. One study looked at correlations between specific dietary components and microbial function and structure in the intestines of a human cohort known for keeping meticulous diet logs (Muegge et al., 2011). They found that there were significant correlations between microbial gene function (Kegg orthologs) and protein intake, confirming the difference that was seen across multiple *mammalian* species between carnivores and herbivores. They also reported a correlation between insoluble fiber consumption and bacterial community membership. A large-scale microbiome sequencing effort called the American Gut Project is currently underway and is attempting to address the effects of diet on the adult microbiome capturing extremes within the American diet (i.e., vegan, paleo, etc) where cultural and environmental factors will be minimized.

### DIETARY INTERVENTIONS INTRODUCE TRANSIENT AND SUBTLE CHANGES IN THE MICROBIOME

Short-term dietary interventions that include introducing novel food components or altering macronutrient levels have also been examined for their effects on intestinal microbial populations. The first of these studies followed obese individuals partitioned to restricted calorie diet groups that controlled for either fat or carbohydrate intake (Ley et al., 2006). Regardless of the macronutrient composition of the diet, individuals that lost a significant amount of body weight had a change in their ratio of Bacteroidetes to Firmicutes, driven by increases in the Bacteroidetes. Weight-loss driven changes in the microbiome was recently confirmed in individuals consuming a calorie restricted liquid diet where it was demonstrated that weight stability of an individual was a better predictor of fecal microbiome stability than time between sample collections (Faith et al., 2013). However, this and another study (Duncan et al., 2008) noted changes in members of the Firmicutes rather than an increase in Bacteroidetes when corresponding weight loss occurred. Calorie restriction in obese and overweight individuals has also been shown to increase microbial gene richness, a parameter that was correlated to improved metabolic parameters (Cotillard et al., 2013; Le Chatelier et al., 2013).

Several studies have noted rapid but transient changes in fecal microbial composition immediately following the start of a dietary intervention study. Wu et al. (2011) conducted a controlled feeding experiment in ten individuals randomized to high fat/low fiber or high fiber/low fat diets and found that although there was no increase in community similarity between individuals on the same diet over a period of 10 days, the first 24 h period was considered an outlier because transient dramatic shifts occurred in the fecal communities of all individuals. Similarly, switching between animal and plant-based diets produces similar results (David et al., 2014). Another interesting finding of the David et al. (2014) study was that foodborne microbes transiently colonized the gut, introducing the idea that food may not only select for commensal bacterial species, but serve as a reservoir for new microbial introductions. Intentional introduction of food-borne microorganisms (probiotics) as well as prebiotic food ingredients and foods high in fiber can also be a means of subtly changing the relative abundance of bacterial species in the gut (Preidis and Versalovic, 2009). Thus, despite the inherent stability of the microbiome over time, changes related to weight loss and diet composition continue to subtly alter the composition and relative abundance of our commensal organisms, driving the development of our gut microbiome throughout adulthood.

## THE AGING GUT

As a person ages, the stability and diversity of their gut microbiota declines with the state of their health. If health remains intact however, microbiota composition often retains the stability and compositional make-up of a healthy younger adult (Claesson et al., 2012). The most prevalent age-related factors influencing the microbial population of the gut are: (1) physiological changes, (2) dietary choices and malnutrition, (3) living situation (community-dwelling, hospitalized, or long-term care), and (4) use of antibiotics (Bartosch et al., 2004; Woodmansey, 2007; Claesson et al., 2012) and other prescription drugs (Qato et al., 2008). This section will explore dietary alterations and antibiotic usage as drivers of change in the elderly gut microbiome and discuss the use of probiotics and prebiotics as potential solutions for the restoration of a healthy gut.

Diet is a major influence on the bacterial makeup of the aging gut. Physiological changes, such as loss of taste and smell, difficulty chewing or swallowing, impaired digestive function, and lack of physical mobility can leave elderly individuals consuming a narrow and nutritionally imbalanced diet, setting the stage for malnutrition (Bartosch et al., 2004; Claesson et al., 2012). Relocation from an in-home community setting to a long-term care facility can change dietary intake as well. The move often contributes to a greater consumption of fat and a decreased intake of fiber, fruits, vegetables, and meat. These dietary alterations are associated with a decrease in microbial diversity and increased frailty (Claesson et al., 2012).

The use of antibiotics in elderly populations is especially prevalent in hospital and long-term care facilities. Antibiotics create an environment of instability by diminishing the population of total and commensal bacteria and opening the door for pathogenic bacteria to overpopulate (Claesson et al., 2011). The use of broad-spectrum antibiotics is associated with the overgrowth of *Clostridium difficile* which flourishes in the antibiotic-weakened gut, often resulting in a life threatening infection (Macfarlane, 2014). As health issues compound and antibiotic use increases, elderly often see a decline in commensal anaerobes (*Bacteroides*, *Lactobacillus* and *Bifidobacterium)* accompanied by a rise in proteolytic and pathogenic bacteria (*Fusobacteria*, *Propionibacteria*, *Clostridia,* and *E. coli*; Wu et al., 2011). Studies indicate that probiotics may have potential as a therapeutic tool to replenish and recolonize beneficial bacterial species like *Bifidobacterium* and *Lactobacillus,* bringing the elderly gut back into balance (Likotrafiti et al., 2014).

### EFFECTS OF DIET AND MALNUTRITION ON THE ELDERLY MICROBIOME

A number of proposed factors contribute to alterations in the elderly gut ecosystem and diet is a significant driver of change (Claesson et al., 2012). Dietary intake can change for a number of reasons with advanced age. Decline in physical mobility may limit access to the grocery store or inhibit the ability to cook. Some elderly lose the desire to eat due to loss of smell and taste or due to slow digestion and prolonged satiety (Britton and McLaughlin, 2013). Malnutrition is often an unintended consequence of age-related physiological changes that can lead to changes in the elderly gut microbiome. Furthermore, studies have shown that compositional dietary changes can result in almost immediate alterations in microbial populations. Wu et al. (2011) found that changes in microbiome composition were detectable within 24 h of dietary alteration and occurred even faster than transit time of food through the gut. In an infant population, malnutrition was shown to delay the maturation of the intestinal microbiota (Subramanian et al., 2014), and it is likely to have consequences of a similar magnitude in the elderly gut.

Dietary changes that come with age are also impacted by living situation. Claesson et al. (2012) found distinct dietary differences between elderly individuals living in a traditional community setting compared to those in long-term care facilities. Community-dwellers may be healthier than their institutionalized counterparts for a number of reasons, but they broadly stated that community-dwellers eat a healthier and more diverse diet and have a distinct microbiota from those in long-term care facilities (Claesson et al., 2012). The largest dietary differences were seen in consumption of fruits, vegetables, and meat. Community-dwellers correlated 98% with a moderate fat/high fiber diet and long-term care dwellers correlated 83% with high fat/low fiber diet (Claesson et al., 2012). The gut microbiota of community-dwellers was more diverse than long-stay subjects and grouped more closely with healthy young adults, indicating that age itself is not the driving factor of microbial change. Similar to young adults, community-dwellers had a higher proportion of phylum Firmicutes and unclassified bacteria, and abundant populations of genera *Coprococcus*, *Roseburia*, *Ruminococcus,* and *Butyricoccus* when compared to long-term stay individuals. Long-stay subjects had a higher incidence of frailty accompanied by a proportional increase in Bacteroidetes and an increased abundance of *Alistipes* and *Oscillibacter* when compared to healthier community-dwelling elderly (Claesson et al., 2012). Increasingly frail individuals showed a significant 26-fold reduction in the number of *Lactobacillus* and a significant sevenfold increase in the number of *Enterobacteriaceae* compared to less frail subjects (van Tongeren et al., 2005).

### ANTIBIOTICS

The compounded effects of poor diet, ailing health, and prolonged stays in a hospital or long-term care facility reduce the prevalence of protective gut microbiota and give way to detrimental populations (Bartosch et al., 2004; Wu et al., 2011). This leaves the elderly individual vulnerable to infection and disease and a prime candidate for antibiotic usage. Unfortunately, antibiotic therapies only exacerbate the flux and instability of the already fragile gut microbiome in unhealthy elderly. The use of antibiotics in elderly populations is especially prevalent in hospital and long-term care facilities and it is estimated that nearly 20% of elderly patients in hospitals are receiving antibiotic treatment at any given time (Bartosch et al., 2004).

Antibiotics cause significant disturbances in gut microbiota resulting in the suppression of both beneficial and pathogenic species, allowing the overgrowth of antibiotic-resistant strains. In young, healthy volunteers administered two separate courses of the antibiotic ciprofloxacin, a dramatic change in the microbiota was noted, followed by the return to an alternative stable state of undetermined consequences (Dethlefsen and Relman, 2011). Use of broad-spectrum antibiotics is associated with the opportunistic bacterium *Clostridium difficile* which flourishes in the antibiotic-weakened gut and results in severe diarrhea (Macfarlane, 2014). Elderly hospital patients and others with fragile immune systems are especially susceptible to this life-threatening infection.

Most often, elderly individuals exposed to antibiotics see an increased relative abundance of Bacteroidetes and a significant increase in Bacteroidetes:Firmicutes ratio (Claesson et al., 2011). Beneficial anaerobic species in the colon such as *Bifidobacterium*, *Lactobacillus*, and *Bacteroides* can be drastically reduced or even eradicated with the use of antibiotics (Bartosch et al., 2004). *Bifidobacterium* and *Lactobacillus* are producers of short chain fatty acids (SCFA’s), a nutrient vital to the proper function of intestinal cells; the loss of these bacteria can be especially detrimental. A study examining the differences in bacterial colonies between healthy elderly, hospitalized patients, and hospitalized patients receiving antibiotics, found that the hospitalized patients receiving antibiotics saw a significant reduction in the numbers of *Bifidobacterium spp*. and an increased relative abundance of *Enterococcus faecalis* compared to the other two groups. In some patients, the antibiotic treatment eliminated certain bacterial communities altogether (Bartosch et al., 2004).

Effects of antibiotic treatment on gut microbiota can differ significantly with the type and dose of antibiotic administered. A study by Bartosch et al. (2004) following elderly patients receiving antibiotics, found that the same antibiotic, clarithromycin, had different effects on gut microbiota at different doses. A low dose of the antibiotic decreased the proportion of Bacteroidetes (*Bacteroides* and *Parabacteroides*) and increased Firmicutes (*Alistipes*) and a high dose increased the proportion of Bacteroidetes (*Parabacteroides*) and decreased the proportion of Firmicutes (*Alistipes*; Claesson et al., 2011). Countless variables must be considered with the use of antibiotics in elderly individuals. What seems like a lifesaving drug may have detrimental effects on the aging microbiome and the health of the individual. Additional research is needed to inform practitioners on the safest ways to use antibiotics on the elderly while supporting their potentially fragile gut microbiota.

### PROBIOTICS AND PREBIOTICS

Probiotics and prebiotics, when taken together or individually, may be particularly beneficial in restoring the proper microbial balance to the elderly gut microbiota, helping to mitigate the detrimental effects of antibiotic usage and under nutrition. Probiotics are live microbes that when administered in sufficient quantities are beneficial to the host. Prebiotics are non-digestible food ingredients such as inulin or various oligosaccharides, which have been show to selectively stimulate growth of beneficial bacterial populations in the large intestine. Probiotic foods and supplements often contain *Bifidobacterium* and/or *Lactobacillus* organisms, both of which are extremely important to proper function of the intestine (Duncan and Flint, 2013). *Bifidobacterium* and *Lactobacillus* are often depleted in elderly individuals as health deteriorates. Research shows that consumption of probiotics containing these strains can result in a notable rise in their abundance along with a reduction of more pathogenic microorganisms in the gut (Toward et al., 2012). Prebiotics may support the *Bifidobacterium* and *Lactobacillus* species delivered via probiotic supplementation by providing a fermentable food source for these bacteria, allowing them to flourish. More specifically, it has been reported that prebiotics have the ability to exert a bifidogenic effect on human subjects (O’Connor et al., 2014).

A recent *in vitro* study showed promise that the elderly gut microbiota can in fact be modulated with appropriate probiotics. Species of *Bifidobacterium* and *Lactobacillus* along with two prebiotics were added to the fecal batch culture of elderly participants. The addition of the beneficial bacteria significantly increased the *Bifidobacterium* and decreased the *Bacteroides* count after fermentation (Likotrafiti et al., 2014). Both probiotic/prebiotic combinations added to the culture increased the *Bifidobacterium* and *Lactobacillus* count in the vessel representing the distal colon. These results represented a major shift in the gut microbiota toward a healthier colon (Likotrafiti et al., 2014). However, prebiotics alone have also been shown to improve the health and alter the gut microbial composition of elderly populations. A study providing inulin supplementation to an elderly cohort increased *Bifidobacterium* levels (Guigoz et al., 2002). Multiple studies using either fructo or GOSs demonstrated both bifidogenic effects and beneficial immune-related effects. Specific immune related effects included reduction in pro-inflammatory cytokines and an increase in the anti-inflammatory cytokine, IL-10.

While probiotic supplementation has become a widely utilized tool to positively impact health by assisting with digestion, bolstering intestinal barrier function and coordinating with the body to regulate both the innate and specific immune responses, the mechanisms by which they exert these beneficial effects is poorly understood (Siciliano and Mazzeo, 2012). Proteomic-based probiotic research is beginning to inform both researchers and industry that adaptation and adherence properties specific to probiotic strains influence their ability to colonize the host (Siciliano and Mazzeo, 2012; van de Guchte et al., 2012). Additionally, these adaptation and adherence mechanisms have been reported to potentially be strain specific, making it difficult to globally apply these mechanisms to all probiotic bacterial strains (Siciliano and Mazzeo, 2012).

Experimentation on the effects of probiotics and prebiotics of the elderly gut microbiome is still limited, but results of the available research lends merit to the notion that beneficial bacteria in the form of probiotics and the indigestible fibers of prebiotics has potential to help restore stability, increase diversity and beneficially alter the immune system in the aging gut (Vulevic et al., 2008). However, these beneficial effects must be placed in perspective given the lack of a mutually agreed upon selection criteria, evaluation methodologies and a clear mechanistic model. With the reduced cost of sequencing and continued proteomic research, hopefully researchers will be able to speak with increased certainty as to the reasons probiotics can be beneficial to human host.

## CONCLUSION

The microbes that reside in our gastrointestinal tract comprise a dynamic community that changes throughout the lifespan of an individual. The early years of infancy and childhood are characterized by a microbial state that has been described as chaotic because of the rapid and dramatic fluctuations observed. While the microbiota of small children begins to resemble that of adults at a very early age, there is a paucity of studies examining temporal microbial community shifts in children beyond infancy, so the stability of their microbiota is not known. Once stable dietary patterns are established, the microbiota of adults remains relatively unaltered; however, significant weight changes have been associated with a higher amount of microbial instability. Finally, factors related to aging, including increased use of pharmaceuticals and changes in diet likely play an important role in shaping the microbial communities residing in the elderly. Changes in physical activity and hormone levels may also be important determinants of the elderly microbiome, but they have not yet been investigated with sufficient depth. Some evidence suggests that the microbial communities of healthy elderly individuals are similar to that of younger adults, but whether the health of the individual contributes to microbial stability or vice versa is not known. Current data suggest that diet is an important driver in the development of the gut microbiome and could serve as a means of therapeutic intervention for prevention of diseases. Studies linking the composition and function of the gut microbiome and disease development certainly highlight the need for a better understanding of temporal microbiome dynamics and their predictors.

## Conflict of Interest Statement

The authors declare that the research was conducted in the absence of any commercial or financial relationships that could be construed as a potential conflict of interest.
